# Tenofovir-silver nanoparticles conjugate ameliorates neurocognitive disorders and protects ultrastructural and cytoarchitectonic properties of the prefrontal cortex in diabetic rats

**DOI:** 10.17305/bjbms.2021.6699

**Published:** 2022-02-05

**Authors:** Sodiq Kolawole Lawal, Samuel Oluwaseun Olojede, Sheu Oluwadare Sulaiman, Okikioluwa Stephen Aladeyelu, Roshila Moodley, Edwin C. Stephen Naidu, Carmen Olivia Rennie, Onyemaechi Okpara Azu

**Affiliations:** 1 Discipline of Clinical Anatomy, School of Laboratory Medicine and Medical Sciences, Nelson R Mandela School of Medicine, University of KwaZulu-Natal, Durban, South Africa; 2 Postgraduate Program in Cell Biology and Birbrair Laboratory, Institute of Biological Sciences, Federal University of Minas Gerais (UFMG), Minas Gerais, Belo Horizonte, Brazil; 3 School of Chemistry and Physics, University of KwaZulu-Natal, Westville Campus, Durban, South Africa; 4 Department of Anatomy, School of Medicine, University of Namibia, Windhoek, Private, Namibia

**Keywords:** Tenofovir, prefrontal cortex, silver nanoparticles, neuroinflammation, neurological dysfunction

## Abstract

Tenofovir disoproxil fumarate (TDF) is the highly recommended antiretroviral drug in human immunodeficiency virus management. Although research has shown the neurological and metabolic disorders associated with TDF administration, the effect of TDF-silver nanoparticles conjugate (TDF-AgNPs) on the disorders has not been fully elucidated. Thus, this study evaluated the neuroprotective effects of TDF-AgNPs on ultrastructural and cytoarchitectonic properties of the prefrontal cortex (PFC) in diabetic rats. Forty-two adult male Sprague-Dawley rats (250 ± 13 g) were randomly divided into non-diabetic groups (1–3) and diabetic groups (4–6), each administered distilled water (0.5 ml/100g, p.o), TDF (26.8 mg/kg/bw, p.o) or TDF-AgNPs (6.7 mg/kg, i.p). After eight weeks of administration, cognitive function, oxidative injury, and tissue inflammation were evaluated. Furthermore, PFC ultrastructure was observed using transmission electron microscopy, Nissl staining, and immunohistochemistry. Diabetic rats administered TDF exhibited cognitive deficits; and increases in blood glucose, malondialdehyde, and interleukin-1 beta (IL-1β) levels, which correlate with decreases in glutathione level, and superoxide dismutase (SOD) and catalase (CAT) activities. Furthermore, loss of PFC astrocytes and neuronal organelles was observed. Conversely, TDF-AgNPs administration to diabetic rats improved cognitive deficits; and increased glutathione, SOD, and CAT, but reduced PFC malondialdehyde and IL-1β concentrations. Notably, TDF-AgNPs prevented loss of PFC neurons and astrocytic cells, and morphology aberration of neuronal organelles. This study suggests that TDF-AgNPs attenuated cognitive deficits via silver nanoparticles’ antioxidant and anti-inflammatory properties, preventing the loss of PFC astrocytes and neurons. The TDF-AgNPs may be utilized to ameliorate the neurological dysfunction caused by prolonged TDF administration.

## INTRODUCTION

The introduction of combined antiretroviral therapy (cART) has a tremendous positive impact in managing patients living with the human immunodeficiency virus (HIV) [1,2]. The cART has multiple benefits, such as suppressing HIV replication, reducing HIV to a manageable level, and improving the quality of life among HIV-positive people [3-5].

Despite the benefits of cART, prolonged usage has been associated with oxidative stress, neuronal injury, and mitochondrial dysfunction leading to neurological and cognitive deficits [6,7].

The standard cART, as a fixed dose, consists of two nucleoside reverse transcriptase inhibitors (NRTIs) with either one of the non-nucleoside reverse transcriptase inhibitors, protease inhibitors, or integrase inhibitors [8]. The NRTIs such as Tenofovir disoproxil fumarate (TDF), emtricitabine, and lamivudine are considered the backbone of cART due to their antiviral efficacy and narrower resistance barrier [9]. TDF is the most recommended NRTIs used in HIV pre-exposure or post-exposure prophylaxis. However, TDF is ranked as a low central nervous system (CNS)-penetration drug considering its physicochemical characteristics, cerebrospinal fluid concentration, and effectiveness in the CNS [9-11]. More so, a 25% reduction in the TDF concentration was observed in dried blood spots of people with diabetes mellitus compared with non-diabetic control (NC) adults [12]. Besides, long-term administration of NRTIs, especially TDF, is associated with metabolic disorders, diabetes mellitus, and systemic and organ toxicity [13,14]. Insulin-like growth factor-1 (IGF-1) is involved in the pathogenesis of metabolic disorders related to diabetes and its complications [15]. Studies have reported the negative impact of TDF on the IGF-1 level in blood and brain samples and its implication in diabetes [16,17].

Furthermore, NRTIs need intracellular anabolic phosphorylation in the host cell to form phosphorylated NRTI metabolites, which have been associated with mitochondrial toxicity and oxidative injury [18,19]. Oxidative neuronal injury, mitochondrial dysfunction, and neuroinflammation have been implicated in neurocognitive deficits [20,21]. The prefrontal cortex (PFC) plays a vital role in cognitive and executive functions, and it is an established brain area to study working memory in animal models [22-24]. Studies have reported that ultrastructural brain injury and up-regulated inflammatory cytokines (e.g., interleukine-1 beta [IL-1β]) were associated with cognitive dysfunction in HIV-positive children on long-term cART [11,25].

Despite the benefits of TDF in HIV management and control, studies have proposed its dose modification in people living with diabetes mellitus and HIV due to its low CNS penetration and neurotoxicity [11,12]. For these reasons, using the nano-delivery system to navigate TDF via the bloodbrain barrier and improving its CNS concentration while minimizing its neurotoxic effects would provide immense therapeutic benefits. Nanoparticles hold promise in delivering antiretroviral drugs due to their ability to cross the biological barrier and long-term release while achieving drug efficacy [26-28]. Silver nanoparticles (AgNPs) are known for their unique properties such as morphology, size, and high surface to volume ratio, making them suitable to be used as intracellular delivery agents [29]. Silver nanoparticles are the most widely used nanoparticles in biomedical sciences as antioxidant and antidiabetic agents [30-32], which may reduce the metabolic and neurotoxic effects of TDF.

Hence, this study aimed at evaluating the therapeutic and neuroprotective effects of Tenofovir-silver nanoparticles conjugate on the cognitive function, and ultrastructural and cytoarchitectonic properties of the PFC in experimental type-2 diabetic rats.

## MATERIALS AND METHODS

### Drugs and chemicals

Enzyme-linked immunosorbent assay (ELISA) kits for IL-1β (Cat No. E-EL-R0012) and IGF-1 (Cat No. E-EL-R0010) were purchased from BIOCOM Africa (pty), Ltd, (South Africa). Streptozotocin (STZ), trisodium citrate (TSC), sodium hydroxide, and silver nitrate (AgNO_3_) were purchased from Sigma-Aldrich (South Africa). Moreover, TDF (TDF, 300 mg) was purchased from Dis-Chem pharmacy Durban, South Africa.

### Experimental animals

Forty-two adult male Sprague-Dawley rats (250 ± 13 g) were obtained from the University of KwaZulu-Natal, Biomedical Research Unit (BRU). The rats were housed in the standard animal laboratory room, maintained at a temperature of 24-26° C, 12:12 light: dark cycle and 40-60% humidity.

### Experimental design

After acclimatization for 7 days, the rats were randomly divided into six groups (n = 7/group) and treated for eight weeks. Groups 1-3 were non-diabetic rats designated as NC administered 0.5 ml/100g distilled water per os (p.o.), non-diabetic + Tenofovir (NT) administered 26.8 mg/kg/bw TDF p.o., and non-diabetic + silver-nanoparticles + tenofovir (NST) administered 6.7 mg/kg TDF-AgNPs intraperitoneally (i.p.). Groups 4-6 were diabetic rats designated as diabetic control (DC) administered 0.5 ml/100g distilled water p.o., diabetic + Tenofovir (DT) administered 26.8 mg/kg/bw TDF p.o., and diabetic + silver-nanoparticles + tenofovir (DST) administered 6.7 mg/kg TDF-AgNPs i.p. The drug dosage was determined according to Everson et al. [33].

### Induction of type 2 diabetes mellitus in rats

Type 2 diabetes mellitus was induced using the fructose-STZ rat model as described by [34]. Briefly, Groups 4-6 rats received 10% fructose solution *ad libitum* for two weeks. On the last day of fructose administration, the rats were fasted overnight, followed by a single intraperitoneal injection of freshly prepared 40 mg/Kgbw STZ dissolved in 0.9% NaCl with 100 mM sodium citrate buffer (pH 4.5) [35]. The control rats received an equal volume of vehicle (citrate buffer). Animals with fasting blood glucose (BG) levels ≥200 mg/dL were considered diabetic and included in this study.

### Formulation of TDF silver nanoparticles (TDF-AgNPs)

Silver nanoparticles (AgNPs) were synthesized according to the method of Turkevich et al. [36]. Briefly, silver nitrate (AgNO_3_) crystals were oven-dried at 100° C. Then, 0.3 g of the AgNO_3_ was weighed into a 500 ml volumetric flask and dissolved with double distilled water to prepare an aqueous solution (0.03 M). The aqueous stock solutions (0.5M, 1M, 1.5M, and 2M) of TSC were prepared from 14.7 g, 29.41 g, 44.12 g, and 58.82 g of TSC in 250 mL of double-distilled water and used as reducing and stabilizing agent. The resultant solution of each of the four different TSC concentrations and AgNO_3_ was continuously stirred for 5 minutes at 90° C, adjusted to pH 10.5 using sodium hydroxide (NaOH), and then stirred for 90 minutes at 90 °C during which color changes were observed from colorless to amber yellow. The synthesized silver nanoparticles (AgNPs) were cooled at room temperature, centrifuged at 12 000 rpm for 15 minutes, and then oven-dried at 40° C for 12 hours.

The TDF silver nanoparticles (TDF-AgNPs) were synthesized by mixing 100 ml of different concentrations (0.5 M, 1 M, 1.5 M, 2 M) of synthesized AgNPs with a 100 ml stock solution of TDF (0.35 M). The final mixture (TDF-AgNPs) was stirred on an ultra-sonicator to ensure proper reaction of TDF and AgNPs. After that, TDF-AgNPs were centrifuged at 4,500 rpm and 40° C, for 40 minutes. The supernatant obtained was analyzed with a UV spectrophotometer at a wavelength of 364 nm to calculate the percentage incorporated efficiency.

TDF-AgNPs percentage incorporated efficiency (% IE) was calculated according to the method of Govender et al. [37] as 
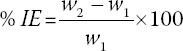
 = 85.00 ± 0.0 %.

W_1_ = quantity of unincorporated drug and W_2_ = total amount of drug coupled with the silver nanoparticles.

The characterization of AgNPs and TDF-AgNPs was done to select the appropriate TSC concentration based on the nanoparticles’ absorbance peaks, size and shape, and morphology of the conjugated TDF-AgNPs. The ultraviolet-visible (UV-Vis) spectroscopy (Shimadzu MultSpec-1501, Shimadzu Corporation, Tokyo, Japan) indicated an absorption peak from 325 to 328 nm. Fourier transform infrared spectroscopy (Perkin-Elmer Universal ATR spectrometer, USA) was used to identify the various functional groups in the TDF-AgNPs conjugates. The functional groups C-N and O-H were present on TDF-AgNPs, indicating that TDF was successfully incorporated into AgNPs.

Furthermore, the high-resolution transmission electron microscope (HR-TEM, JEOL 2100, Japan) operated at a voltage of 200 kV showed spherical particles for 2 M of TDF-AgNPs, and the particle size was between 12 nm and 22 nm.

The field emission scanning electron microscope (FESEM, Carl Zeiss, Germany) operated at a voltage of 5 kV with energy dispersive X-ray (EDX, Aztec Analysis Software, England), and the EDX was used to determine the elemental components. This analysis revealed the presence of silver, sodium, and other functional groups from AgNPs and TDF, indicating that the silver nanoparticles were successfully incorporated with TDF.

### Blood glucose (BG) level

The blood samples were obtained through the tail vein, and the BG measurement was determined using a portable glucometer (Sigma-Aldrich, Durban, South Africa).

### Behavioral assessment

The Y maze test was used to measure the experimental rats’ cognitive functions (working memory) and explorative behaviors. The “spontaneous alternation performance” and “same arm return (SAR)” was done following previously used methods [38,39]. Briefly, Y-maze comprises three equal arms, each measuring 40 cm long, 15 cm high, and 10 cm wide, and interconnected with one another at 120°. The arms were identified as A, B, and C. Each rat started the experiment at the center of the maze and was allowed to move freely for 5 minutes. The whole body must completely enter the arm from the center area before recording is taken as one entry. The number of entries was recorded using a video camera, and two independent observers recorded the data from the computer system.

Spontaneous Alternative Performance (SAP) and SAR were analyzed, and the percentage alternation was calculated as previously reported [39]:

% Alternation = (Number of Alternations/[Total number of arm entries-2]) × 100).

### Neurochemical analysis

#### Preparation of brain homogenates

The brain tissue was harvested and immediately rinsed in cold phosphate-buffered saline (PBS), and the PFC was dissected on the ice tray. PFC (0.5 g) was weighed and homogenized in 5 mL of sodium phosphate buffer (0.1M, pH 7.5). The homogenates were centrifuged for 10 mins at 20,000 g, and the supernatants were obtained for neurochemical analyses.

#### Determination of superoxide dismutase (SOD) and catalase (CAT) activities, and reduced glutathione (GSH) and malondialdehyde (MDA) levels

PFC tissue homogenates were analyzed for SOD and CAT activities as well as GSH and MDA levels by spectrophotometric assay. SOD and CAT activities were determined following the methods of Kakkar et al. [40] and Aebi [41], respectively. Reduced GSH level was assessed using the Ellman protocol [42]. MDA level was determined by measuring the content of thiobarbituric acid reactive products using the method of Mkhwanazi et al. [43].

### Analysis of inflammatory biomarkers

The concentrations of cytokine IL-1β and IGF-1 were quantified in the PFC homogenates using their specific ELISA kits (Elabscience Biotechnology Co., Ltd., Houston, TX, USA) according to the manufacturer’s instructions.

### Brain tissue processing for microscopic study

The brains were carefully removed from the rat’s skull and then postfixed in 10% neutral buffer formalin (NBF) for 1 hour. It was then transferred to 15% sucrose in PBS (24 hours) followed by 30% sucrose in PBS for 12 hours and finally fixed in 10% NBF for histology and immunochemistry through paraffin embedding. The PFC tissues were sectioned at 5 μm using Leica RM 2255 microtome, followed by clearing in xylene, hydration in descending grades of alcohol, Nissl staining according to [44], and mounting with dibutyl phthalate polystyrene xylene [45].

### Immunohistochemical (IHC) analysis

The PFC was used for the IHC analysis of Glial Fibrillary Acidic Protein (GFAP). The sections were washed in PBS 2 times for 10 minutes each (2 × 10 minutes) at 4° C and pre-incubated in 0.1 M PBS. This process was followed by 5% normal goat serum with 0.4% Triton X-100 and 1% bovine serum albumin for one hour at 4° C. The process was then followed by a direct incubation in the GFAP primary antibody (anti-GFAP) diluted in the phosphate-buffered Sodium Azide (PBSA)-Triton (PBSAT). After that, tissue sections were incubated in 0.1 M PBS containing 2% normal goat serum and biotinylated rabbit anti-goat IgG (secondary antibody) (1:2000) for 2 hours at room temperature. The sections were then rinsed in PBSAT (2 × 10 minutes) and then incubated with the avidin-biotin complex (AB; 1:2000) for 2 hours at room temperature, followed by several washes (1 × 10 minutes in PBST and 2 × 10 minutes in Tris buffer [0.05 M, PH 7.6]). The peroxidase activity detection was carried out with 3-3’ diaminobenzidine (DAB, 0.025%, 0.5% Nickel ammonium sulphate in tris buffer (0.1 M, pH 7.6) with 0.03% hydrogen peroxide. After that, the immunoreactive reaction was stopped by washing the sections once in 0.1 M Tris buffer (10 minutes) and then twice in 0.1 M PBS (10 minutes). Sections were dehydrated in ascending grades of ethanol baths, cleared in two successive xylene baths, mounted onto gelatin-coated slides, and covered slipped with Eukitt.

### Ultrastructural tissue processing

The brain tissues were initially fixed in buffered 2.5 % glutaraldehyde for 12 hours, washed in phosphate buffer (3 × 5 minutes), and postfixed in 1 % osmium tetroxide for 2 hours. This was followed by washing in phosphate buffer (3 × 5 minutes), dehydrating in ascending grades of Acetone (30%, 50%, 75%, and 100%) for 5 minutes each, and embedding in Durcopan (Fluka). Ultrathin sections of PFC were cut using an ultramicrotome, contrasted by uranyl acetate and lead acetate, and the prepared tissue sections were examined by transmission electron microscopy (TEM).

### Ethical statement

All rats were handled following standard guides for the animal laboratory and were allowed free access to water and feed *ad libitum*. The experimental protocols were approved by the Animal Ethics Committee (AREC) of the University of KwaZulu-Natal (AREC/044/019D).

### Statistical analysis

Data were presented as mean ± SEM. The differences between means were compared using one-way analysis Analysis of variance, followed by Tukey’s multiple comparisons test. All analyses were done using GraphPad Prism 8 for Windows (GraphPad Software San Diego, CA 92108). *p* < 0.05 was considered statistically significant.

## RESULTS

### TDF-AgNPs reduces BG and PFC IGF-1 levels

The results in Table 1 show that there was a significant (*p* < 0.05) increase in BG level in the DC group (DC) compared with the non-diabetic group (NC). In non-diabetic groups, there was an increased BG level in TDF treated group (NT) (89.26 ± 26) compared with NC (80.86 ± 2.65). Notably, the non-diabetic group treated with TDF-AgNPs (74.26 ± 1.52) had a significant reduction (*p* < 0.05) in the BG compared with TDF treated only.

**TABLE 1:**
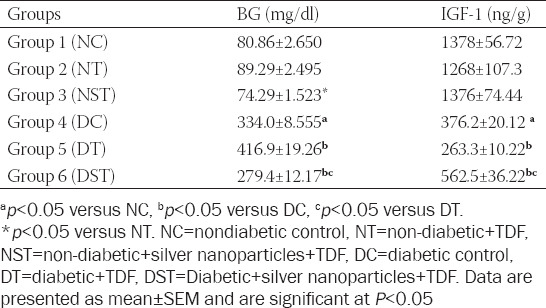
Blood glucose and prefrontal cortex Insulin-like growth factor-1 levels

Diabetic rats treated with TDF (group DT) (416.9 ± 19.26) had a significant increase (*p* < 0.05) in BG level compared with DC (334.0 ± 8.6). Interestingly, the rats administered with TDF-AgNPs (DST) (279.4 ± 12.17) had a significant decrease (*p* < 0.05) in BG level compared with DC and diabetic + TDF group (DT).

In non-diabetic groups, TDF-treated group NT (1268 ± 107.3) had a decrease in IGF-1 compared with NC rats (NC) (1378 ± 56.72) and group NST (TDF-AgNPs) (1376 ± 74.44). The DC group (DC) (376.2 ± 20.12) had a significant (*p* < 0.05) decrease in IGF-1 compared to the NC group (NC). IGF-1 was significantly decreased in group DT (diabetic + TDF) (263.3 ± 10.22) compared with group DC. However, group DST (diabetic + AgNPs+ TDF) (562.5 ± 36.22) had a significant increase in IGF-1 compared with group DC and group DT.

### TDF-AgNPs improves cognitive functions using Y-maze

As shown in Table 2, there was a significant decrease in spontaneous alternation performance (a measure of cognitive performance) and an increase in SAR in all diabetic groups (DC, DT, and DST) compared with NC. Furthermore, there was a significant decrease in SAP in diabetic rats treated with TDF (group DT) (37.50 ± 2.239) compared with DC (51.01 ± 2.205). However, the administration of TDF-AgNPs to diabetic rats significantly improved the SAP (62.39 ± 2.265) compared with DC (51.01 ± 2.205) and diabetic rats treated with TDF only (37.50 ± 2.239). The diabetic rats treated with TDF-AgNPs (group DST) (28.57± 0.7190) had a significant decrease in the percentage of SAR compared to groups DC (37.29 ± 1.554) and DT (38.43 ± 2.181).

**TABLE 2:**
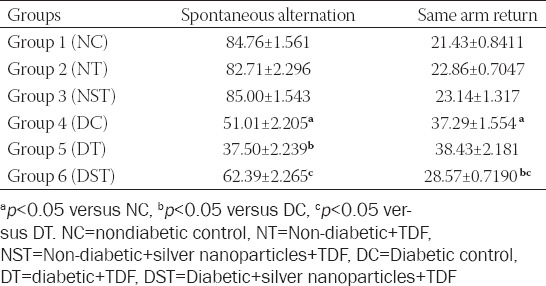
Y maze parameters (SAP and SAR) for cognitive functions

### TDF-AgNPs alleviates oxidative stress

**FIGURE 1:**
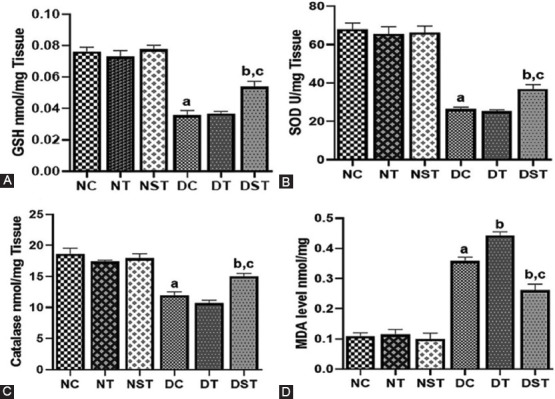
Effect of TDF-AgNPs on oxidative stress markers (GSH, SOD, CAT and MDA). (A) p < 0.05 vs NC, (B) p < 0.05 v DC, (C) p < 0.05 v DT. NC=nondiabetic control, NT=non-diabetic + TDF, NST=non-diabetic+ silver nanoparticles + TDF, DC= diabetic control, DT=diabetic + TDF, DST= diabetic +silver nanoparticles+ TDF, A= Reduced glutathione, B= superoxide dismutase (SOD), C = catalase (CAT), Reduced glutathione D= malondialdehyde (MDA).

As presented in Figure 1, there was a significant (*p* < 0.05) increase in MDA but a decrease in GSH, SOD, and CAT in the diabetic group (DC) compared with NC. The diabetic rats treated with TDF (group DT) had no significant difference in GSH, SOD, and CAT compared with DC. However, MDA was significantly increased in group DT (10.71 ± 0.47) compared with the DC (11.94 ± 0.58). Notably, the diabetic rats treated with TDF-AgNPs (group DST) **(**15.04 ± 04**)** had a significant (*p* < 0.05) decrease in MDA level compared with the DC group (11.94 ± 0.58). Also, there was a significant increase in GSH, SOD, and CAT in group DST compared with group DT.

### TDF-AgNPs reduces PFC IL-1β

**FIGURE 2:**
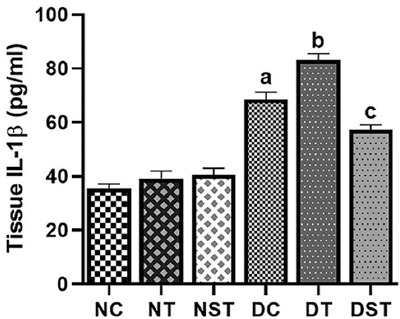
Effect of TDF-AgNPs on interleukin-1 beta (IL-1β). ap < 0.05 vs NC, bp < 0.05 v DC, cp < 0.05 v DT. NC=nondiabetic control, NT=non-diabetic + TDF, NST=non-diabetic+ silver nanoparticles + TDF, DC= diabetic control, DT=diabetic + TDF, DST= diabetic +silver nanoparticles+ TDF, interleukin-1 beta (IL-1β).

As presented in Figure 2, the concentration of IL-1β (an inflammatory marker) was significantly (*p* < 0.05) increased in DC (group DC) (68.550 ± 2.69) compared to NC (group NC) (35.48 ± 1.77). Group DT showed a significant (*p* < 0.05) increase in IL-1β (83.24 ± 2.33) compared with group DC (68.550 ± 2.69). Notably, administration of TDF-AgNPs to diabetic rats showed a significant improvement in IL-1β compared (57.32 ± 1.80) with diabetic rats treated with TDF only (83.24 ± 2.33).

### TDF-AgNPs improves GFAP positive astrocytes of the PFC

**FIGURE 3:**
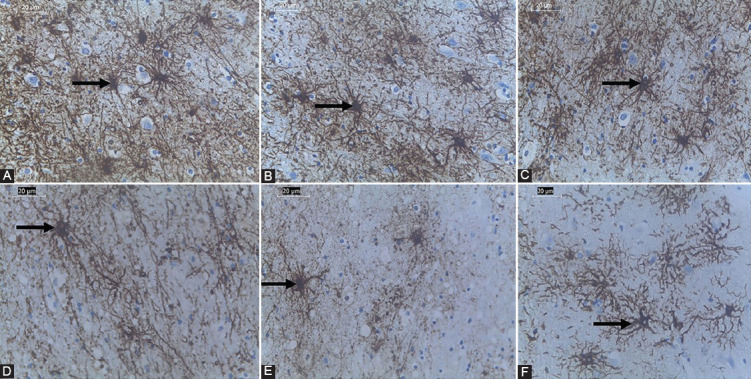
Effect of TDF-AgNPs on PFC-GFAP positive astrocytes. (A) nondiabetic control (NC), (B) non-diabetic + TDF (NT), (C) non-diabetic+ silver nanoparticles + TDF (NST), (D) diabetic control (DC), (E) diabetic + TDF (DT), (F) diabetic +silver nanoparticles+ TDF (DST). Black arrow indicates GFAP positive astrocyte.

As shown in (Figure 3A-F), there was a significant (*p* < 0.05) decrease in PFC-GFAP positive astrocytes in DC (20.28 ± 0.55) (DC, Figure 3D) compared with NC (40 ± 2.13) (NC). The diabetic rats treated with TDF only (group DT, Figure 3E) had a significant (*p* < 0.05) reduction in PFC-GFAP positive astrocytes (19 ± 1.52) compared with DC (20.28 ± 0.55) (DC). Interestingly, group DST (Diabetic + TDF-AgNPs) had an improved number of PFC-GFAP positive astrocytes (29.17 ± 0.89) (Figure 3F) compared with group DC and group DT (diabetic + TDF).

### TDF-AgNPs improves ultrastructure of neuronal mitochondrial and glial cells

**FIGURE 4:**
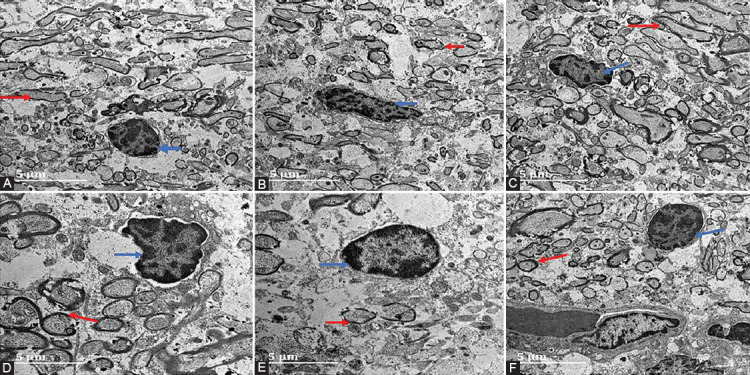
Effect of TDF-AgNPs on PFC neuronal mitochondrial and glial cells. (A) nondiabetic control (NC), (B) non-diabetic + TDF (NT), (C) non-diabetic+ silver nanoparticles + TDF (NST), (D) diabetic control (DC), (E) diabetic + TDF (DT), (F) diabetic +silver nanoparticles+ TDF (DST). Blue Arrow indicates neuronal mitochondrial and red arrow indicates glial cells.

As shown in (Figure 4A-F), the neuronal mitochondria in group NC (non-diabetes control) appeared normal with a round shape, an intact mitochondrial membrane, and numerous surrounding myelin sheaths (Figure 4A). The non-diabetic rat treated with TDF (group NT) showed an elongated mitochondrion (Figure 4B). Vacuolation and prominent mitochondria were seen in DC (group DC, Figure 4D) compared with group NC. Diabetic rats treated with TDF show few myelin sheaths with disrupted and prominent mitochondria (Figure 4E). Notably, group DST (Diabetes+ TDF-AgNPs) showed round-shaped mitochondria with thick and smooth edges of the myelin sheath (Figure 4F).

### TDF-AgNPs preserves PFC pyramidal cells

**FIGURE 5:**
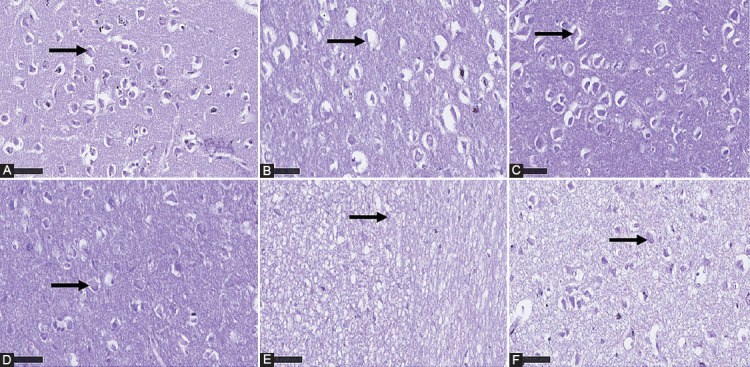
(A-F) Effect of TDF and TDF-AgNPs on the prefrontal cortex pyramidal cells using Nissl staining in non-diabetic and diabetic rats. (A) nondiabetic control (NC), (B) non-diabetic + TDF (NT), (C) non-diabetic + silver nanoparticles + TDF (NST), (D) diabetic control (DC), (E) diabetic + TDF (DT), (F) diabetic + silver nanoparticles + TDF (DST). Black arrow indicates pyramidal cells Nissl stain x 800, 50 μm.

The effect of TDF and TDF-AgNPs on the PFC pyramidal cells in non-diabetic and diabetic rats is presented in (Figure 5 A-F). The PFC in all non-diabetic groups (Figure 5A-C) shows deep Nissl staining of the pyramidal cells. The DC group (Figure 5D) showed necrotic pyramidal cells and poorly stained Nissl bodies. The diabetic rats treated with TDF only had poor Nissl staining outcomes and scanty pyramidal cells with weak affinity for Nissl stain. Notably, the diabetic rats treated with TDF-AgNPs show improved Nissl staining characteristics of PFC pyramidal cells (Figure 5F).

## DISCUSSION

This study evaluated the benefits of tenofovir-silver nanoparticles conjugate on the ultrastructural and cytoarchitectonic properties of the PFC in type-2 diabetic rats. TDF is commonly used as pre-exposure prophylaxis alone or in combination with other antiretroviral drugs as post-exposure prophylaxis [46,47]. Despite TDF’s low CNS-penetration rate, studies have reported its neurotoxic effect, diabetic and neurocognitive implications [11,12,19]. Silver nanoparticles have been shown to cross the bloodbrain barrier independent of dosage and route of administration [48,49]. In addition, silver nanoparticles possess antidiabetic and antioxidant effects and long-term drug release ability, which may be suitable for delivering TDF to brain tissues.

This study showed a significant increase in BG in diabetic rats treated with TDF only. Incidences of insulin resistance and hyperglycemia have been reported in the long-term use of NRTIs. Thus, they are associated with type-2 diabetes mellitus [50,51]. However, a study indicated that TDF alone has less risk of insulin resistance [52]. Administration of TDF to diabetic rats in this study caused a significant negative impact on PFC IGF-1. Further decrease in IGF-1 observed in diabetic rats treated with TDF compared with DC rats indicated that TDF exacerbated hyperglycemia in diabetic rats by reducing the IGF-1 level. Previous studies have stated the negative effect of diabetes and TDF on IGF-1 levels in blood and brain samples [16,17]. Notably, TDF-AgNPs treated diabetic rats showed a decrease in BG level that corresponded with a significant improvement in IGF-1. The improvement in glycaemic status and IGF-1 level in diabetic rats treated with TDF-AgNPs may be due to antidiabetic and antioxidant effects of silver nanoparticles in the conjugates via scavenging the free radicals caused by TDF and diabetes [53,54]. In addition, silver nanoparticles have been shown to influence glycaemic control by enhancing hepatic glycogenesis via the insulin signaling pathway and increasing serum insulin concentration [55]. These factors may contribute to the blood-glucose-lowering effects observed in the diabetic rats treated with TDF-AgNPs.

Furthermore, MDA significantly increased in diabetic rats treated with TDF only, correlating with hyperglycemia. Chronic hyperglycemia has been linked to increased free radical production and oxidative stress [56]. More so, TDF has been particularly implicated in mitochondrial toxicity [57].

Interestingly, tenofovir-silver nanoparticles conjugate (TDF-AgNPs) alleviated the oxidative stress in the diabetic rats via reducing the MDA while increasing the antioxidant enzymes (GSH, SOD, and CAT). This finding suggests that silver nanoparticles’ antioxidant property may alleviate free radical damage caused by diabetes and TDF while promoting the antioxidant enzymes in diabetic rats. Studies have attributed antioxidant properties of AgNPs to inhibition of reactive oxygen species production and improved activities of antioxidant enzymes, thereby scavenging free radicals [53,58].

Further investigation on diabetes and TDF treatment showed an increase in the PFC inflammatory cytokine level. An increase in free radical and oxidative injury compromises the integrity of the PFC tissue, which may account for the elevated IL-1β and its neuroinflammatory effects. The diabetic TDF-treated rats had a significant reduction in GFAP positive astrocytes and an increase in mitochondrial damage with few myelin sheaths on ultrastructural assessment. These results corroborate the report of Zulu and colleagues [7], who observed up-regulation of IL-1β, brain tissue injury, and neurocognitive disorder in experimental rats treated with TDF.

The consequences of the PFC tissue damage via oxidative injury and neuroinflammation were obviously seen during the behavioral assessment in this study. TDF caused a decrease in spontaneous alternation performance in diabetic rats while the SAR s significantly increased. This observation indicates a neurocognitive disorder and supports the findings of Zulu *et al*. [7], who reported that TDF promotes neuroinflammation and contributes to the neurocognitive disorder.

In this study, tenofovir-silver nanoparticles (TDF-AgNPs) reduced the neuroinflammatory marker (IL-1β) and preserved the PFC-GFAP positive astrocytes and neuronal organelles. Consequently, these neuroprotective effects were correlated with improved neurobehavioral activities. Oxidative injury and neuroinflammation of PFC tissue are implicated in neurocognitive disorders [59]. Notably, TDF-AgNPs preserved some of the pyramidal cells of the PFC in the diabetic rats compared with the diabetic TDF-treated only rats. An increase in Nissl staining of the pyramidal cells in this group suggested that some of the Nissl bodies were protected by the antioxidant properties of silver nanoparticles. More so, the overall improvement in the PFC cytoarchitectures was associated with improved cognitive functions.

Silver nanoparticles act as anti-inflammatory and antioxidant agents, protecting the neuronal cell against diabetes and TDF-induced oxidative injury [55,60]. More so, a recent study has reported the advantage of using silver nanoparticles for tissue restoration and regeneration [30,60]. This indicates that an improvement in the neurocognitive functions observed in the tenofovir-silver nanoparticles conjugate treated rats may be attributed to the potency of silver nanoparticles to restore or delay neuronal cell injury. Moreover, TSC used as a reducing and stabilizing agent in the preparation of silver nanoparticles has been reported to influence the toxicity profile by reducing Ag^+^ to Ag^0^ in the conjugates [61,62]. In addition, the size and shape of nanoparticles are determining factors in their biological system interaction and organ toxicity [63]. In this study, the characterization of TDF-AgNPs revealed nanoparticles between 20 and 35 nm with a spherical shape. The observed size and shape may influence the positive impact of silver nanoparticles observed in TDF-AgNPs treated diabetic rats. Previous animal studies showed no adverse effects and organ toxicity in chemically synthesized small-medium silver nanoparticles between 20 nm and 50 nm [64,65].

## CONCLUSION

This study suggests that TDF-AgNPs attenuated cognitive deficits via antioxidant and anti-inflammatory properties of silver nanoparticles by preventing the loss of astrocytes and neurons of the PFC. This drug formulation may be used to ameliorate neurological dysfunction caused by prolonged administration of TDF, HIV-associated neurological disorders, and neglected ART-induced neurological deficits in managing HIV infection.
